# Risk assessment of urban yellow fever virus transmission in Kenya: is *Aedes aegypti* an efficient vector?

**DOI:** 10.1080/22221751.2022.2063762

**Published:** 2022-05-05

**Authors:** Sheila B. Agha, David P. Tchouassi, Michael J. Turell, Armanda D.S. Bastos, Rosemary Sang

**Affiliations:** a International Centre of Insect Physiology and Ecology, Nairobi, Kenya; b Department of Zoology and Entomology, University of Pretoria, Hatfield, South Africa; c VectorID LLC, Frederick, MD, USA; d Arbovirus/Viral Hemorrhagic Fever Laboratory, Center for Virus Research, Kenya Medical Research Institute (KEMRI), Nairobi, Kenya

**Keywords:** *Aedes aegypti*, *Aedes bromeliae*, vector competence, yellow fever virus, urbanization, East Africa, transmission barrier

## Abstract

The absence of urban yellow fever epidemics in East Africa remains a mystery amidst the proliferation of *Aedes aegypti* in this region*.* To understand the transmission dynamics of the disease, we tested urban (Mombasa, Kisumu, and Nairobi) *Aedes* mosquito populations in Kenya for their susceptibility to an East African yellow fever virus (YFV) genotype. Overall, 22% (*n* = 805) of the *Ae. aegypti* that were orally challenged with an infectious dose of YFV had a midgut infection, with comparable rates for Mombasa and Kisumu (χ^2 ^= 0.35, df = 1, *P* = 0.55), but significantly lower rates for Nairobi (χ^2^ ≥ 11.08, df = 1, *P* ≤ 0.0009). Variations in YFV susceptibility (midgut infection) among *Ae. aegypti* subspecies were not associated with discernable cytochrome c oxidase subunit 1 gene haplotypes. Remarkably, no YFV dissemination or transmission was observed among the orally challenged *Ae. aegypti* populations. Moreover, *Ae. aegypti* mosquitoes that were intrathoracically inoculated with YFV failed to transmit the virus via capillary feeding. In contrast, dissemination (oral exposure) and transmission (intrathoracic inoculation) of YFV was observed among a few peri-domestic *Ae. bromeliae* mosquitoes (*n* = 129) that were assessed from these urban areas. Our study highlights an inefficient urban *Ae. aegypti* population, and the potential for *Ae. bromeliae* in sustaining an urban YFV transmission in Kenya. An assessment of urban *Ae. aegypti* susceptibility to other YFV genotypes, and vector potential of urban *Ae. bromeliae* populations in Kenya is recommended to guide cost-effective vaccination.

## Introduction

Despite being preventable by vaccine, yellow fever (YF) has re-emerged in the past decade as one of the major public health challenges, with fatality rates as high as 40% recorded among confirmed cases [1]. YF is currently endemic in sub-Saharan African and South American countries [2,3], and up to seven genotypes of the disease-causing pathogen (yellow fever virus, YFV) are in circulation [4]. As the burden of YF is projected to shift from West Africa to East and Central Africa by 2050 [5], measures that can guide cost-effective vaccination, through the identification of at-risk populations, are paramount for the successful implementation of the Eliminate YF Epidemics strategy in these regions [6].

YF has three transmission cycles in Africa (urban, intermediate or rural, and the sylvatic or jungle), with each cycle involving a specific set of vertebrate hosts and vectors [4,7–9]. Urban YF epidemics mediated by *Aedes aegypti* are the most dreaded due to the potential for rapid spread, in densely populated areas, placing largely naïve and unvaccinated populations at risk [6]. Urban YF has predominantly been reported in West African countries whereas sylvatic YF is observed in other endemic African regions, including East Africa [4,10]. Recent urban YF outbreaks in Luanda (Angola, Southern Africa) and Kinshasa (Democratic Republic of the Congo, Central Africa) [11] signal a shift in YF epidemiology for these regions, from the sylvatic to the urban transmission cycles, yet with limited understanding of vector potential and unknown epidemiological consequences for East Africa.

Sylvatic YF epidemics in East Africa are increasing both in magnitude and frequency with recent epidemics reported from Sudan (2012), South Sudan (2018, 2020), Uganda (2010–2011, 2016, 2019–2020), and Ethiopia (2012–2014, 2018, 2020) [12–16]. Kenya witnessed a sylvatic YF outbreak in 1992/1993, which was caused by peri-domestic and sylvatic mosquito species such as *Ae. africanus*, *Ae. kinenesis*, and potentially also *Ae. bromeliae* [17]. The rapid expansion of the makeshift nature of urban areas with limited sanitary provision in Kenya has encouraged the concentration of susceptible human hosts and the proliferation of mosquito vectors making urban areas increasingly prone to massive outbreaks. The porous nature of Kenya’s borders along with increasing rural–urban migration presents opportunities for YF to be introduced into urban Kenya from neighbouring East African countries experiencing outbreaks, and the potential for emergence of YFV from the sylvatic into the urban cycle. International travel presents an additional risk of local arbovirus transmission that could be initiated by the importation of cases from endemic regions [18], as occurred when two YF cases were imported into Kenya (port of entry and location of patient treatment, city of Nairobi) from Angola during the 2015–2016 outbreak [19]. Local transmission may well have been initiated had both patients not been post-viremic on arrival.

Local arbovirus transmissions are propagated in the presence of an infectious virus, susceptible human hosts, competent vectors, and a permissive environment [20]. In our previous YF risk assessment study, we identified *Ae. aegypti* and *Ae. bromeliae* (in much lower abundance relative to *Ae. aegypti*) as the two potential YFV vectors present in urban Kenya [21,22]. Without a prior knowledge of the ability of these urban vectors to transmit YFV (vector competence), we estimated the risk of urban YF emergence in Kenya as low-medium, a conclusion that was guided by established vector indices with threshold levels set according to WHO guidelines [23]. A risk assessment study is, however, not complete without a detailed understanding of key vector parameters, including vector competence and genetics. Virus susceptibility is believed to vary between *Ae. aegypti* subspecies [24,25] although this remains poorly characterized especially for YFV. Here, we present findings on the vector competence of urban *Aedes* species for transmitting YFV. We additionally assessed whether the genetic variability existing within the dominant vector, *Ae. aegypti*, may explain variable susceptibility to YFV.

## Materials and methods

### Ethical statement

Scientific and ethical approval for the study was obtained from Kenya Medical Research Institute Scientific and Ethics Review Unit (KEMRI-SERU) (Project Number SERU 2787). The animal use component was reviewed and approved (approval number KEMRI/ACUC/ 03.03.14) by the KEMRI Animal Use and Care Committee (KEMRI ACUC). The KEMRI ACUC adheres to national guidelines on the care and use of animals in research and education in Kenya enforced by the National Commission for Science, Technology and Innovation (NACOSTI). The institute has a foreign assurance identification number F16-00211 (A5879-01) from the Office of Laboratory Animal Welfare (OLAW) under the Public Health Service and commits to the International Guiding Principles for Biomedical Research Involving Animals.

### Mosquito collection and rearing

Populations of *Ae. aegypti* mosquitoes were collected from selected sites in three urban areas of Kenya: Kisumu (Kanyarkwar and Kajulu), Mombasa (Rabai, outskirts Kilifi), and Nairobi (Githogoro). These study sites have previously been described (see Figure 1 in Ref. 21 for map of study sites) [21]. Immature *Ae. aegypti* and *Ae. bromeliae* were collected from water holding containers indoor and outdoor and from natural larval habitats like leaf axils. Mosquitoes were collected between April and November 2016 and transported to the enhanced BSL-2 insectary at the Kenya Medical Research Institute (KEMRI) set at 28°C and 12:12 h (L:D) photoperiod for rearing. The larvae were fed on tetramine fish food (Tetramin) and the emerging adults were morphologically identified [26,27] as *Ae. aegypti* or *Ae. bromeliae* and placed in separate cages on the basis of species and sampling locality. *Aedes bromeliae* were used as F_0_ (we did not succeed to hatch the eggs collected from *Ae. bromeliae* under laboratory conditions) for all subsequent experiments. Adult *Ae. aegypti* were fed on 6% glucose solution supplied on cotton wool and females were blood-fed using anesthetized laboratory mice (KEMRI, Animal House) to stimulate egg development. Eggs were collected as described previously [28] and the resulting F_1_ & F_2_ adults were used for the vector competence experiment.

### Yellow fever virus amplification

An East African YFV genotype, isolated from a patient during the 1992/1993 YF outbreak in Kerio Valley, Kenya, was used in this study. The virus stock had previously been passaged once in a suckling mice brain and twice on Vero cells (Green African Monkey cell line, ATTC® CCL-81) and stored at −80°C. Prior to use in this study, an additional passage was done on Vero cells, cultured in cell culture media consisting of Minimum Essential Media (MEM) (Sigma-Aldrich, St. Louis, MO) as previously described [28,29]. Viral amplification was achieved by inoculating 600 μl of the virus suspension with a titre 10^3.5^ plaque-forming units (PFU)/ml on freshly cultured Vero cells in a T-75 cell culture flask (Corning Incorporated, USA). Following a 1-h incubation (in a 5% CO_2_ incubator set at 37°C) with intermittent rocking to allow for virus adsorption, the virus-infected cells were maintained in 20 ml maintenance media (MEM, supplemented with 2% FBS). The cells were incubated and observed daily, and once 80% cytopathic effect (CPE) was observed the contents of the flask were frozen at −80°C. The following day, the contents of the flask were gently thawed on wet ice and centrifuged (Eppendorf centrifuge 5417R) at 1500 rpm for 5 min at 4°C. The supernatant (titre 10^7.5^ PFU/ml) was aliquoted into 1.5 ml cryotubes and either used immediately or stored at −80°C as virus stock for later use in this study.

### Infection, dissemination, and transmission assays for orally exposed mosquitoes

An infectious blood meal was prepared by adding two parts of defibrinated sheep blood (Central Veterinary Laboratories Kabete, Kenya) to one part of freshly harvested or frozen YFV in separate experiments. Mouse skin was used as a membrane to cover the wells of a hemotek membrane feeder (Discovery Workshops, Accrington, UK). The YFV infectious blood was introduced into the well of the feeding system (2 ml per well) maintained at 37°C. In three replicates, female *Ae. aegypti* mosquitoes, 5–12 days old, pre-starved for 12 h, were allowed to feed for 1 hour. Before and after exposure of the mosquitoes, 100 µl of the infectious blood was added to 900 µl of homogenization media (MEM, supplemented with 15% FBS) to determine the virus titre before and after feeding. The blood/media mixtures were immediately stored at −80°C until virus quantification via cell culture techniques.

Fully engorged mosquitoes were aspirated into new cages and incubated in an insectary set at 28°C, 12:12 h (L:D) photoperiod, for up to 21 days. Mosquitoes were maintained on 6% glucose delivered via cotton wool ad libitum. A proportion of mosquitoes exposed to the freshly cultured YFV were analysed for the presence of virus on days 7, 14, and 21. Individually, the legs and wings of immobilized mosquitoes were removed, the body was placed on a sticky tape and the proboscis was inserted into a capillary tube containing 15–20 μl of homogenization media for 30 min to collect saliva [29]. The legs, body, and saliva (emptied into a microcentrifuge tube containing 150 µl homogenization media) samples were immediately frozen in separate microcentrifuge tubes at −80°C for subsequent virus testing. The body and legs samples were then triturated in 500 µl homogenization media and assayed for virus infection and dissemination, respectively, via cell culture techniques. The saliva-containing samples were assayed for virus transmission [28,29]. Because no virus dissemination or transmission was observed for mosquitoes exposed to a freshly cultured YFV, subsequent mosquitoes exposed to the frozen YFV stock were not tested for virus transmission.

### Intrathoracic inoculation of mosquitoes

To more efficiently assess transmission by a mosquito with a disseminated infection, 5–12-day-old *Ae. aegypti* mosquitoes from the different urban areas were individually inoculated intrathoracically with 0.3 µl of a YFV suspension [30]. The suspension was prepared by adding 100 µl of the YFV stock (containing 10^7.5^ PFU/ml) to 900 µl of homogenization media. After 10–14 days of incubation at 28°C, individual mosquitoes were immobilized and the body (head, thorax, abdomen, and legs) and saliva samples were collected and analysed for virus as done for the orally exposed mosquitoes. Inoculation bypasses tissue barriers [31] and thus, this group of mosquitoes was not used to calculate the infection or dissemination rates.

### Virus assays

The titre of the blood/virus mixture was determined by plaque assay as previously described [28]. Briefly, 10-fold serial dilutions (up to 10^−4^) of the individual samples were prepared and inoculated (100 µl per well) on freshly cultured 80% confluent Vero cells in a 6-well plate. The cells were overlaid with 2 ml of 2.5% methylcellulose (mixed with 2X MEM) and the plates incubated. After 9 days, the cells in each well were fixed with 10% formalin for 2 h and then stained with 0.5% crystal violet overnight. Plaques were observed and counted using a light box. Individual mosquito samples were triturated and centrifuged as previously described [29]. For each sample, 50 µl of the supernatant was inoculated and cultured in a single well (on a 24-well plate) containing 80% confluent Vero cells. Following incubation at 37°C, the plates were observed daily for cytopathic effect for up to 12 days (CPE assay). The supernatant of wells showing CPE were harvested and frozen down at −80°C. These were subsequently re-tested to confirm the growth of infectious virus particles. About 25% of the negative samples (body and legs) were also re-tested using a CPE assay to confirm the results. The saliva-containing samples were processed in the same manner. Virus recovery from the body, legs, and saliva confirmed infection, dissemination, and transmission of YFV, respectively.

### Molecular phylogenetic analysis

To assess whether the genetic variability existing within *Ae. aegypti* has an effect on vector competence, we compared the genetics of individual mosquitoes between susceptible and non-susceptible cohorts after exposure to the virus. This was achieved by sequence analysis of the barcoding region of the mitochondrial cytochrome c oxidase subunit 1 (*COI*) gene. This marker has proven useful to differentiate *Ae. aegypti* lineages representing the subspecies [29,32]. Genomic DNA was extracted from the legs of individual *Ae. aegypti* mosquitoes with- and without a midgut infection (i.e. YFV positive and negative body samples) from each of the three populations to determine the subspecies. DNA extraction was performed using the DNeasy Blood and Tissue Kit (Qiagen, GmbH-Hilden, Germany) per the manufacturer’s instructions. Briefly, DNA amplification targeted the *COI* barcoding region using *COI* FOR (5’-TGTAATTGTAACAGCTCATGCA-3’) and REV (5’-AATGATCATAGAAGGGCTGGAC-3’) primers for *Ae. aegypti* subspecies identification [32]. Amplicons of the expected size (860 bp) were individually purified using ExoSap PCR purification kit (Thermo Fisher Scientific), according to recommendations by the manufacturer. Unidirectional sequencing using the forward primer was outsourced to a commercial firm (Macrogen, Seoul, Republic of Korea) and sequences were viewed and edited in Chromas, prior to phylogenetic analysis using MEGA v 5 software [33]. Homologous sequences in the Genbank database were identified through BlastN searches and aligned using ClustalW in MEGA. Reference *COI* sequences for domestic *Ae. aegypti* (Genbank Accession No. MF194022 and No. AF390098) and *Ae. aegypti formosus* (Genbank Accession No. AY056597) were included. The best-fit model of sequence evolution identified under the Bayesian Information Criterion was used to infer a Maximium Likelihood (ML) tree in MEGA v 5 and guided the selection of priors for Bayesian inference (BI) with MrBayes 3.2 [34]. Nodal support was assessed through 1000 bootstrap replications for ML and from Bayesian posterior probabilities obtained from two independent runs of 10 million generations each, with burn-in set to 25%, for the BI analyses. The haplotypes generated in this study were deposited in GenBank under accession numbers OL963781-OL963831.

### Data analysis

Virus infection and dissemination were ascertained by confirming the presence of the virus in the mosquito’s body (head, thorax, and abdomen) and legs, respectively. Mosquitoes with a positive body but negative legs were considered to have a non-disseminated infection limited to the midgut. If both the body and legs were positive, the mosquito was considered to have a disseminated infection [28]. Also, a positive saliva indicated virus transmission potential. The virus infection rates of *Ae. aegypti* from the different areas were compared using the Chi-squared test. All analyses were performed in R version 3.3.1 [35] at *α* = 0.05 level of significance.

## Results

### *Aedes aegypti* susceptibility to oral infections with the yellow fever virus

The YFV titres of the blood meals to which mosquitoes were exposed were determined to be 10^6.0^ to 10^6.5^ PFU/ml for the freshly cultured virus (range indicates titre before and after infectious blood meal) and 10^5.8^ to 10^6.2^ PFU/ml for the frozen virus. A total of 805 mosquitoes from Mombasa (*n* = 267), Kisumu (*n* = 328), and Nairobi (*n* = 210) were exposed to an infectious blood meal containing YFV virus (Table 1). Overall, mosquitoes exposed to freshly grown YFV had higher infection rates compared to those exposed to frozen YF, although the difference was not significant (Table 1). Because no significant difference was observed the overall infection rates for each city were combined. No significant difference in the overall infection rates was observed between Mombasa and Kisumu (χ^2 ^= 0.35, df = 1, *P* = 0.55), but infection rates were significantly lower when mosquitoes from Nairobi were compared to those from Mombasa (χ^2 ^= 11.08, df = 1, *P* = 0.0009), and to Kisumu (χ^2 ^= 16.30, df = 1, *P* < 0.0001).

**Table 1.** Susceptibility of *Aedes aegypti* mosquitoes from Mombasa, Kisumu, and Nairobi to oral infection with yellow fever virus.

		Percent infected^a^ (No. infected/No. tested) by days post-exposure to yellow fever virus			
Area	State of the virus	7	14	21	Total
Nairobi	Frozen	14 (10/70)	7 (4/56)	11 (4/34)	11 (18/160)
	Freshly cultured	13 (2/15)	20 (3/15)	10 (2/20)	14 (7/50)
	Total	14 (12/85)	9 (7/71)	11 (6/54)	12 (25/210)
Kisumu	Frozen	22 (21/92)	27 (24/87)	28 (24/84)	26 (69/263)
	Freshly cultured	25 (5/20)	45 (9/20)	20 (5/25)	29 (19/65)
	Total	23 (26/112)	30 (33/107)	26 (29/109)	26 (88/328)
Mombasa	Frozen	25 (20/78)	30 (19/63)	14 (10/68)	23 (49/209)
	Freshly cultured	26 (4/15)	40 (6/15)	21 (6/28)	27 (16/58)
	Total	25 (24/93)	32 (25/78)	16 (16/96)	24 (65/267)

^a^ Infection rate (No. infected/No. tested * 100).

Analysis of mosquitoes on days 7, 14, and 21 post-exposure to the infectious blood meal indicated that infection rates were not significantly different by day, post-virus exposure (Table 1). The leg samples of all the mosquitoes with infected bodies (resulting from the batch of mosquitoes that were orally exposed to the freshly cultured *n* = 42 and frozen *n* = 136) tested negative for the YFV. This confirmed the absence of YFV dissemination. Similarly, the saliva samples of all mosquitoes that were orally exposed to the freshly cultured YFV all tested negative.

### *Aedes bromeliae* susceptibility to oral infections with the yellow fever virus

We only tested *Ae. bromeliae* populations from Mombasa and Nairobi. Insufficient samples were collected from Kisumu despite several collection attempts. The overall infection rate recorded for *Ae. bromeliae* was 19% and the dissemination rate was 3% (17% dissemination rate for mosquitoes with a midgut infection) for all the orally exposed specimens. The infection and disseminiation rates in Mombasa and Nairobi were not significantly different (*P* ≥ 0.05). Unlike *Ae. aegypti,* dissemination was observed for *Ae. bromeliae,* albeit only after 14 days post-exposure to the virus for both populations (Table 2). Transmission rates were, however, not assessed for this group of mosquitoes.

**Table 2.** Susceptibility of *Aedes bromeliae* mosquitoes from Mombasa and Nairobi to oral infection with yellow fever virus (YFV).

	Percent infected^a^ (No. infected/No. tested) by days post-exposure to YFV				Percent disseminated^b^ (No. disseminated/No. tested) by days post-exposure to YFV				
Area	7	14	21	I.R.^c^	7	14	21	D.R.^d^	D.R.(I)^e^
Nairobi	11 (2/18)	12 (3/26)	17 (5/30)	14 (10/74)	0 (0/18)	4 (1/26)	3 (1/30)	3 (2/74)	20 (2/10)
Mombasa	0 (0/14)	21 (4/19)	48 (10/21)	26 (14/54)	0 (0/14)	5 (1/19)	5 (1/21)	4 (2/54)	14 (2/14)
Total	6 (2/32)	16 (7/45)	29 (15/51)	19 (24/128)	0 (0/32)	4 (2/45)	4 (2/51)	3 (4/128)	17 (4/24)

^a^ Infection rates (No. infected/No. tested * 100).

^b^ Dissemination rate (No. disseminated/No. tested * 100).

^c^ Total infection rate (total No. infected/total No. tested * 100).

^d^ Total dissemination rate (total No. disseminated/total No. tested * 100).

^e^ Total dissemination rate for infected mosquitoes (total No. disseminated/total No. infected * 100).

Although no virus dissemination was observed for the *Ae. aegypti* mosquitoes in this study, the *Ae. aegypti* population in Nairobi (χ^2 ^= 0.024, df = 1, *P* = 0.89) and Mombasa (χ^2 ^= 0.005, df = 1, *P* = 0.94) did not appear to be any less susceptible to the virus at the level of the midgut (infection rates), when compared to *Ae. bromeliae* populations from the same area.

### Yellow fever virus susceptibility among intrathoracic inoculated *Aedes aegypti* mosquitoes

High mortality rates were observed in the mosquitoes within 48 h of inoculation which was most likely due to the inoculation process. After 10–14 days post inoculation, the bodies (head, thorax, abdomen, and legs) of all 44 surviving inoculated *Ae. aegypti* mosquitoes tested positive while, all the saliva samples tested negative for the YFV indicating a salivary gland barrier (Table 3). The only surviving inoculated *Ae. bromeliae* mosquito was infected and transmitted YFV through its saliva collected using the capillary feeding method.

**Table 3.** Susceptibility level of *Aedes* mosquitoes inoculated intrathoracically with yellow fever virus.

Area	Mosquito species	Percent infected^a^ (No. positive/No. tested)	
		Body	Saliva
Nairobi	*Ae. aegypti*	100 (12/12)	0 (0/12)
Kisumu	*Ae. aegypti*	100 (16/16)	0 (0/16)
Mombasa	*Ae. aegypti*	100 (16/16)	0 (0/16)
Mombasa	*Ae. bromeliae*	100 (1/1)	100 (1/1)

^a^ Percent infected (No. positive/No. tested * 100).

### Genetic diversity of *Aedes aegypti* mosquitoes with and without a midgut infection

**Figure 1.**
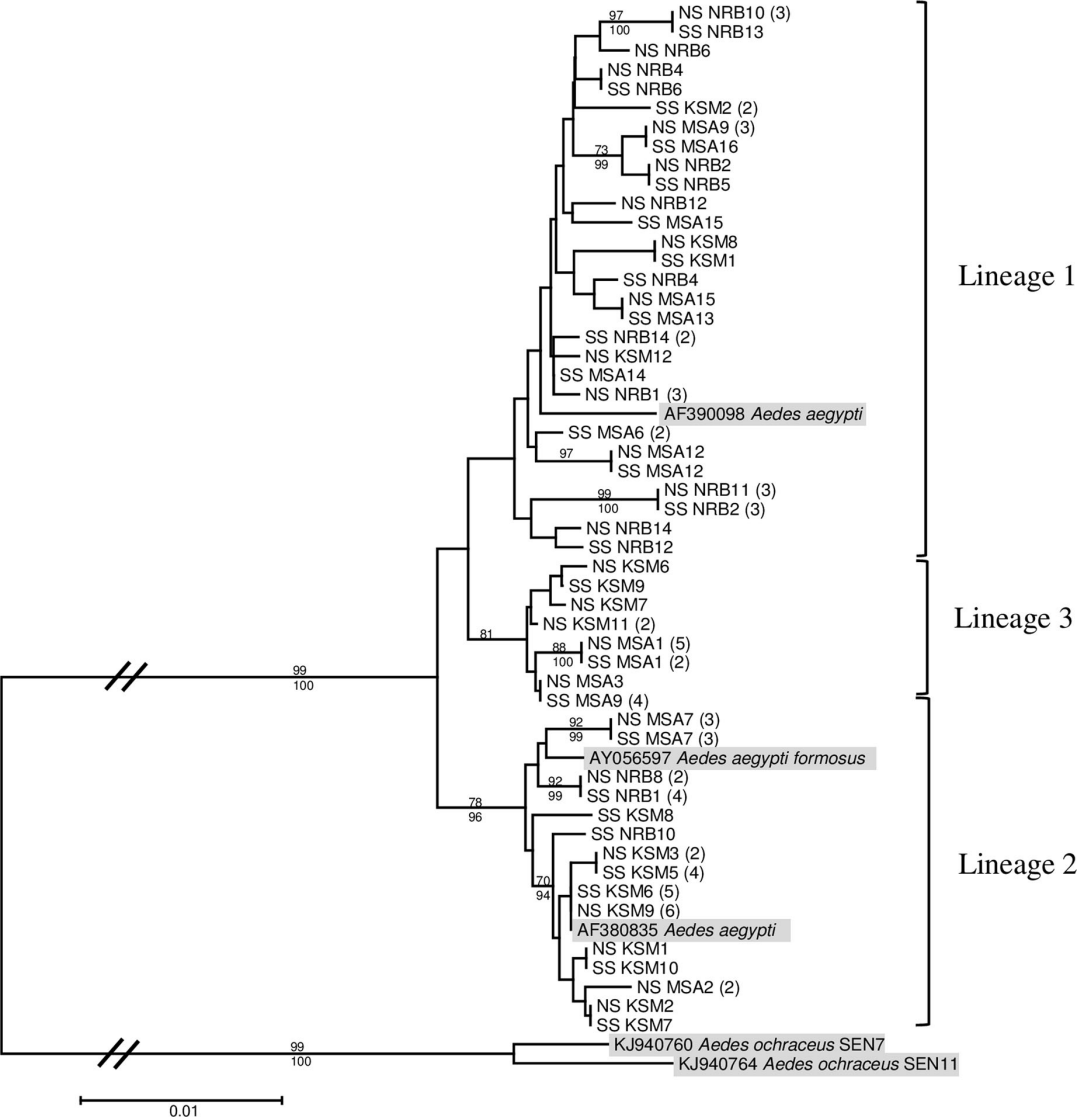
Maximum likelihood tree inferred using the (T92 + G + I) model of sequence evolution for *COI* barcode region (860 bp) of yellow fever midgut infected (SS) and non-infected (NS) *Ae. aegypti* samples from Nairobi (NRB), Kisumu (KSM), and Mombasa (MSA), Kenya. The number of individuals sharing a haplotype is indicated in parentheses. Bootstrap support values from 1000 replications ≥65 and Bayesian posterior probabilities ≥90 are indicated above and below the three major lineages, respectively, with terminal nodes reflecting bootstrap support values alone. *Aedes ochraceus* was included as an outgroup.

*Aedes aegypti* mosquitoes with and without a midgut infection from all three urban areas were randomly selected for phylogenetic analysis. We recovered three lineages: Lineage 1 clustered closely with the anthropophilic *Ae. aegypti aegypti* (Genbank Accession No. AF390098), Lineage 2 with a zoophilic *Ae. aegypti formosus* (Genbank Accession No. AY056597 and AF380835), and a third lineage within a well-supported clade (81% bootstrap support) distinct from both the anthropophilic and zoophilic *Ae. aegypti* lineages (Figure 1). Observed infection rates amongst the various clades were all similar, indicating that virus susceptibility was not associated with any of the three *COI* gene lineages recovered.

## Discussion

As part of an urban YF risk assessment study, we set out to assess the vector competence of domestic/peri-domestic *Aedes* mosquitoes from the three major cities in Kenya in transmitting YFV. Our results showed that urban *Ae. aegypti* populations in Kenya were unable to disseminate or transmit an East African YFV genotype while *Ae. bromeliae* did. This observation could be attributed to probable tissue barriers or innate immune responses that have previously been reported to limit virus transmission within arbovirus vectors [36]. Phylogenetic analysis additionally confirmed that the genetic variability existing within *Ae. aegypti* populations does not appear to influence vector competence and the epidemiology of urban YF in Kenya.

*Aedes aegypti* populations from three urban areas (Nairobi, Kisumu, and Mombasa) in Kenya failed to disseminate or transmit an East African YFV genotype within 21 days post-oral viral exposure. Virus dissemination out of the mosquito midgut and subsequent replication in secondary tissues is key for a successful virus transmission [37]. Because the blood meal to which the mosquitoes were exposed contained a titre (10^6^ PFU/ml) similar to that of a viremic person, the inability of this mosquito species to sustain a virus replication beyond the midgut epithelial tissues was indicative of potentially inefficient urban vector populations. Our laboratory observation for Nairobi concurs with a previous report of an inefficient urban *Ae. aegypti* population in Kenya when, vector susceptibility to a YFV isolated from Sudan, 2003, was assessed [38]. We did not assess vector competence beyond 21 days post-oral viral exposure or hold mosquitoes at a higher temperature. However, the daily survival of *Ae. aegypti*, an important factor in vectorial capacity, needs to be considered [39]. Also, we did not measure the viral load in the bodies of the infected/non-virus disseminating *Ae. aegypti* mosquitoes, yet, this could improve our understanding of how the viral load varies with time post-virus exposure and partly explain the phenotypic observations. Future studies should consider this.

The observed absence of YFV dissemination (absence of the virus in mosquito legs) and transmission among orally exposed *Ae. aegypti* implied a significant midgut escape barrier in these populations of *Ae. aegypti*. Mosquito leg tissues are routinely used to ascertain virus dissemination [28,29,40–42]. The presence of a significant midgut escape barrier in *Ae. aegypti* could potentially prevent viral particles from infecting the hemocoel, a prerequisite for salivary gland infection [36]. Future studies investigating intrinsic factors [43,44] limiting YFV replication within *Ae. aegypti* should be investigated using F_0_ mosquitoes. This could provide new insights into YFV transmission-blocking strategies within the mosquito thereby enhancing disease control. The absence of virus transmission, even among inoculated *Ae. aegypti*, implied a significant salivary gland (infection or escape) barrier.

To our knowledge, this is the first study linking YFV transmission to the molecular genetics of *Ae. aegypti* subspecies. Previous studies on YFV and *Ae. aegypti* either did not consider intraspecific variation [45] or subspecies were defined by morphology alone [46] rather than molecular methods which are generally regarded to be more reliable. Phylogenetic analysis of the *COI* gene showed that both the anthropophilic and ancestral subspecies of *Ae. aegypti* (*Ae. aegypti aegypti* and *Ae. aegypti formosus*, respectively) were included in our vector competence experiments, and that susceptibility to YFV infections appeared to be similar in both groups. However, although the different genetic forms analysed in this study were equally susceptible to YFV at the level of the midgut, YFV failed to be disseminated from the midgut (leg tissue analysis) or be transmitted by bite (collected saliva analysis) in any of these, or a third *Ae. aegypti* clade, indicating both a significant midgut escape and salivary gland barrier in the *Ae. aegypti* populations tested. In a previous study, rural *Ae. aegypti* populations in Kenya were reported to disseminate (head tissue analysis) YFV, although their ability to transmit the virus was not assessed [38]. The ability of *Ae. aegypti* to sustain a YFV transmission, especially in rural areas, should therefore not be ignored. Additional vector competence studies and a detailed phylogenetic analysis comparing the genetic structure of urban and rural *Ae. aegypti* populations in Kenya should be carried out to understand a possible genetic influence of the populations on susceptibility to YFV. In addition to *COI* other markers including single nucleotide polymorphism markers [45] should be explored to characterize further the genetic diversity of populations of this species in relation to YFV susceptibility.

YFV currently has seven genotypes (East African genotype, West African genotypes I and II, East and Central African genotype, Angola genotype, and two genotypes in South America) circulating in endemic areas in sub-Saharan Africa and South America [4]. Although this study reports no virus dissemination or transmission for *Ae. aegypti*, it is important to note that only the susceptibility to an East African YFV genotype isolated from Kenya was assessed. Would the results be different if a different YFV genotype or a different strain of an East African genotype (e.g. one isolated from Uganda) was used? A prior study of *Ae. aegypti* mosquitoes from West Africa demonstrated that vector competence was flavivirus species and viral genotype dependent [46]. It is therefore possible that the same may apply to this urban *Ae. aegypti* populations in Kenya which failed to disseminate or transmit YFV, yet was previously shown to disseminate and transmit the dengue virus [29]. Also, *Ae. aegypti* mosquitoes from these urban areas have been previously reported to transmit the chikungunya virus under similar laboratory conditions [28]. Additional vector competence studies to assess the susceptibility of urban *Ae. aegypti* populations in Kenya to the other YFV genotypes is thus, highly recommended. This will guide our understanding of the potential risk of transmission that could result from importation in Kenya of a non-endemic genotype.

Unlike *Ae. aegypti*, virus infection, dissemination, and transmission were detected among the few *Ae. bromeliae* assessed in this study*. Aedes bromeliae,* the predominant vector in the *Ae. simpsoni* complex, is a peri-domestic vector well documented as a major YF vector in East Africa [10,47]. *Aedes bromeliae* was implicated in sylvatic YF outbreaks in Ethiopia and Uganda [10] and was suspected to have been involved in a YF outbreak in Kenya [17]. A laboratory vector competence study has previously confirmed that this vector is more efficient than *Ae. aegypti* in transmitting YFV [38]. Although present in low abundance, our previous vector surveillance study confirmed the presence of *Ae. bromeliae* in the major cities of Kenya [21,22]. The ability of *Ae. bromeliae* to initiate local urban YFV transmission from an imported case should therefore not be ignored. The peri-domestic vector, *Ae. bromeliae,* also has the capacity to act as a bridge vector moving YFV from the sylvatic/rural into the urban transmission cycle. A detailed entomological YF risk assessment study including the vectorial capacity of *Ae. bromeliae* is highly recommended in both rural and urban settings in Kenya. Also, unlike *Ae. aegypti* mosquitoes that were assessed as F_1_–F_2_ in our vector competence experiments, *Ae. bromeliae* were assessed as F_0._ The microbiota composition and potential co-infection of F_0_ *Ae. bromeliae* with insect-specific viruses may have modulated the YFV susceptibility observed for *Ae. bromeliae* [48,49]*.* However, more conclusive research on this is needed.

We conclude that in the populations of *Ae. aegypti* evaluated, the presence of major midgut escape and salivary gland barriers indicate that these populations are unlikely to serve as efficient vectors of East African genotypes of YFV. Additional investigations of the vector potential of the peri-domestic vector, *Ae. bromeliae*, in Kenya is recommended as virus dissemination and transmission was observed among the few samples that were assessed from these urban areas. Vector competence remains an important component of risk assessment while continuous vector surveillance and control measures should be performed routinely. Additional risk assessment studies, especially using other circulating YFV genotypes, are also recommended.

## References

[1] Kraemer MUG, Faria NR, Reiner RC, et al. Spread of yellow fever virus outbreak in Angola and the Democratic Republic of the Congo 2015–16: a modelling study. Lancet Infect Dis. 2017;17(3):330–338. 28017559 10.1016/S1473-3099(16)30513-8 PMC5332542

[2] Mathai D, Vasanthan AG. State of the globe: yellow fever is still around and active! J Glob Infect Dis. 2009;1(1):4–6. 20300379 10.4103/0974-777X.52975 PMC2840945

[3] World Health Organisation. Yellow fever. WHO. 2017 [cited 2019 Dec 24]. Available from: https://www.who.int/news-room/fact-sheets/detail/yellow-fever.

[4] Mutebi J-P, Barrett ADT. The epidemiology of yellow fever in Africa. Microbes Infect. 2002;4(14):1459–1468. 12475636 10.1016/s1286-4579(02)00028-x

[5] Gaythorpe KA, Hamlet A, Cibrelus L, et al. The effect of climate change on yellow fever disease burden in Africa. eLife. 2020;9:e55619. 32718436 10.7554/eLife.55619 PMC7386919

[6] World Health Organisation. A global strategy to eliminate yellow fever epidemics (EYE) 2017–2026. Geneva: WHO; 2018 [cited 2021 Oct 1]. 54 p. Available from: https://apps.who.int/iris/handle/10665/272408.

[7] Monath TP. Yellow fever: an update. Lancet Infect Dis. 2001;1(1):11–20. 11871403 10.1016/S1473-3099(01)00016-0

[8] Monath TP, Vasconcelos PFC. Yellow fever. J Clin Virol. 2015;64:160–173. 25453327 10.1016/j.jcv.2014.08.030

[9] Rogers DJ, Wilson AJ, Hay SI, et al. The global distribution of yellow fever and dengue. Adv Parasitol. 2006;62:181–220. 16647971 10.1016/S0065-308X(05)62006-4 PMC3164798

[10] Ellis BR, Barrett ADT. The enigma of yellow fever in East Africa. Rev Med Virol. 2008 Oct;18(5):331–346. 18615782 10.1002/rmv.584

[11] World Health Organisation. Angola grapples with worst yellow fever outbreak in 30 years. WHO; 2016 [cited 2021 Dec 24]. Available from: https://www.who.int/emergencies/disease-outbreak-news/item/6-april-2016-yellow-fever-kenya-en.

[12] Kwagonza L, Masiira B, Kyobe-Bosa H, et al. Outbreak of yellow fever in central and southwestern Uganda, February–May 2016. BMC Infect Dis. 2018;18(1):548. 30390621 10.1186/s12879-018-3440-y PMC6215607

[13] Markoff L. Yellow fever outbreak in Sudan. N Engl J Med. 2013;368(8):689–691. 23387798 10.1056/NEJMp1300772

[14] Mulchandani R, Massebo F, Bocho F, et al. A community-level investigation following a yellow fever virus outbreak in South Omo zone, South-West Ethiopia. PeerJ. 2019;7:e6466. 30809451 10.7717/peerj.6466 PMC6387579

[15] Wamala JF, Malimbo M, Okot CL, et al. Epidemiological and laboratory characterization of a yellow fever outbreak in northern Uganda, October 2010–January 2011. Int J Infect Dis. 2012;16(7):e536–e542. 22575876 10.1016/j.ijid.2012.03.004

[16] World Health Organisation. Disease Outbreak News. 2021 [cited 2021 Oct 5]. Available from: https://www.who.int/emergencies/disease-outbreak-news/4.

[17] Reiter P, Cordellier R, Ouma JO, et al. First recorded outbreak of yellow fever in Kenya, 1992-1993. II. Entomologic investigations. Am J Trop Med Hyg. 1998;59(4):650–656. 9790447 10.4269/ajtmh.1998.59.650

[18] Sousa CA, Clairouin M, Seixas G, et al. Ongoing outbreak of dengue type 1 in the autonomous region of Madeira, Portugal: preliminary report. Eurosurveillance. 2012;17(49):20333. 23231893 10.2807/ese.17.49.20333-en

[19] World Health Organisation. Disease outbreak news, yellow fever – Kenya. WHO; 2016 [cited 2018 May 11]. Available from: https://www.who.int/emergencies/disease-outbreak-news/item/6-april-2016-yellow-fever-kenya-en.

[20] Esser HJ, Mögling R, Cleton NB, et al. Risk factors associated with sustained circulation of six zoonotic arboviruses: a systematic review for selection of surveillance sites in non-endemic areas. Parasit Vectors. 2019;12(1):265. 31133059 10.1186/s13071-019-3515-7 PMC6537422

[21] Agha SB, Tchouassi DP, Bastos ADS, et al. Assessment of risk of dengue and yellow fever virus transmission in three major Kenyan cities based on *Stegomyia* indices. PLoS Negl Trop Dis. 2017;11(8):e0005858. 28817563 10.1371/journal.pntd.0005858 PMC5574621

[22] Agha SB, Tchouassi DP, Bastos ADS, et al. Dengue and yellow fever virus vectors: seasonal abundance, diversity and resting preferences in three Kenyan cities. Parasit Vectors. 2017;10:628. 29284522 10.1186/s13071-017-2598-2 PMC5747025

[23] World Health Organisation. Technical guide for a system of yellow fever surveillance; 1971 [cited 2016 Dec 5]. Available from: http://apps.who.int/iris/bitstream/10665/218621/1/WER4649_493-500.PDF.

[24] Sylla M, Bosio C, Urdaneta-Marquez L, et al. Gene flow, subspecies composition, and dengue virus-2 susceptibility among *Aedes aegypti* collections in Senegal. PLoS Negl Trop Dis. 2009;3(4):e408. 19365540 10.1371/journal.pntd.0000408 PMC2663788

[25] Vazeille-Falcoz M, Failloux AB, Mousson L, et al. Oral receptivity of *Aedes aegypti formosus* from franceville (Gabon, Central Africa) for type 2 dengue virus. Bull Soc Pathol Exot 1990. 1999 Dec;92(5):341–342. 10690473

[26] Edwards FW. Mosquitoes of the Ethiopian region III. – *Culicine* adults and pupae. London: Printed by order of the Trustees, British Museum (Natural History); 1941.

[27] Jupp PG. Mosquitoes of Southern Africa. Hartbeespoort: Ekogilde Publishers; 1996.

[28] Agha SB, Chepkorir E, Mulwa F, et al. Vector competence of populations of *Aedes aegypti* from three distinct cities in Kenya for chikungunya virus. PLoS Negl Trop Dis. 2017;11(8):e0005860. 28820881 10.1371/journal.pntd.0005860 PMC5576749

[29] Agha SB, Tchouassi DP, Turell MJ, et al. Entomological assessment of dengue virus transmission risk in three urban areas of Kenya. PLoS Negl Trop Dis. 2019;13(8):e0007686. 31442223 10.1371/journal.pntd.0007686 PMC6728053

[30] Rosen L, Gubler D. The use of mosquitoes to detect and propagate dengue viruses. Am J Trop Med Hyg. 1974;23(6):1153–1160. 4429185 10.4269/ajtmh.1974.23.1153

[31] Turell MJ, O’Guinn ML, Dohm DJ, et al. Vector competence of North American mosquitoes (Diptera: Culicidae) for West Nile virus. J Med Entomol. 2001;38(2):130–134. 11296813 10.1603/0022-2585-38.2.130

[32] Paupy C, Le Goff G, Brengues C, et al. Genetic structure and phylogeography of *Aedes aegypti*, the dengue and yellow-fever mosquito vector in Bolivia. Infect Genet Evol. 2012;12(6):1260–1269. 22522103 10.1016/j.meegid.2012.04.012

[33] Tamura K, Peterson D, Peterson N, et al. MEGA5: molecular evolutionary genetics analysis using maximum likelihood, evolutionary distance, and maximum parsimony methods. Mol Biol Evol. 2011;28(10):2731–2739. 21546353 10.1093/molbev/msr121 PMC3203626

[34] Ronquist F, Teslenko M, van der Mark P, et al. MrBayes 3.2: efficient Bayesian phylogenetic inference and model choice across a large model space. Syst Biol. 2012;61(3):539–542. 22357727 10.1093/sysbio/sys029 PMC3329765

[35] The R core Team version 3.2.3. R: A language and environment for statistical computing. Vienna: R Foundation for Statistical Computing; 2012.

[36] Franz AWE, Kantor AM, Passarelli AL, et al. Tissue barriers to arbovirus infection in mosquitoes. Viruses. 2015;7(7):3741–3767. 26184281 10.3390/v7072795 PMC4517124

[37] Hardy JL, Houk EJ, Kramer LD, et al. Intrinsic factors affecting vector competence of mosquitoes for arboviruses. Annu Rev Entomol. 1983;28:229–262. 6131642 10.1146/annurev.en.28.010183.001305

[38] Ellis BR, Sang RC, Horne KM, et al. Yellow fever virus susceptibility of two mosquito vectors from Kenya, East Africa. Trans R Soc Trop Med Hyg. 2012;106(6):387–389. 22521217 10.1016/j.trstmh.2012.02.007

[39] Kramer LD, Ebel GD. Dynamics of flavivirus infection in mosquitoes. Adv Virus Res. 2003;60:187–232. 14689695 10.1016/s0065-3527(03)60006-0

[40] Chepkorir E, Lutomiah J, Mutisya J, et al. Vector competence of *Aedes aegypti* populations from Kilifi and Nairobi for dengue 2 virus and the influence of temperature. Parasit Vectors. 2014;7(1):1–8. 25223760 10.1186/1756-3305-7-435 PMC4261593

[41] Lutomiah JL, Koka H, Mutisya J, et al. Ability of selected Kenyan mosquito (Diptera: Culicidae) species to transmit West Nile virus under laboratory conditions. J Med Entomol. 2011;48(6):1197–1201. 22238879 10.1603/me11062

[42] Turell M, Gargan T, Bailey C. Replication and dissemination of Rift Valley fever virus in *Culex pipiens*. Am J Trop Med Hyg. 1984;33(1):176–181. 6696176 10.4269/ajtmh.1984.33.176

[43] Jupatanakul N, Sim S, Dimopoulos G. The insect microbiome modulates vector competence for arboviruses. Viruses. 2014;6(11):4294–4313. 25393895 10.3390/v6114294 PMC4246223

[44] Sim S, Jupatanakul N, Dimopoulos G. Mosquito immunity against arboviruses. Viruses. 2014;6(11):4479–4504. 25415198 10.3390/v6114479 PMC4246235

[45] Tabachnick WJ, Wallis GP, Aitken TH, et al. Oral infection of *Aedes aegypti* with yellow fever virus: geographic variation and genetic considerations. Am J Trop Med Hyg. 1985 Nov;34(6):1219–1224. 3834804 10.4269/ajtmh.1985.34.1219

[46] Dickson LB, Sanchez-Vargas I, Sylla M, et al. Vector competence in West African *Aedes aegypti* is flavivirus species and genotype dependent. PLOS Negl Trop Dis. 2014;8(10):e3153. 25275366 10.1371/journal.pntd.0003153 PMC4183443

[47] Kamau WW, Sang R, Ogola EO, et al. Survival rate, blood feeding habits and sibling species composition of *Aedes simpsoni* complex: implications for arbovirus transmission risk in East Africa. PLoS Negl Trop Dis. 2022;16(1):e0010171. 35073317 10.1371/journal.pntd.0010171 PMC8812930

[48] Patterson EI, Villinger J, Muthoni JN, et al. Exploiting insect-specific viruses as a novel strategy to control vector-borne disease. Curr Opin Insect Sci. 2020 Jun;39:50–56. 32278312 10.1016/j.cois.2020.02.005 PMC7302987

[49] Hegde S, Rasgon JL, Hughes GL. The microbiome modulates arbovirus transmission in mosquitoes. Curr Opin Virol. 2015;15:97–102. 26363996 10.1016/j.coviro.2015.08.011 PMC5731638

